# Translational potential of synaptic alterations in Alzheimer’s disease patients and amyloid precursor protein knock-in mice

**DOI:** 10.1093/braincomms/fcad001

**Published:** 2023-01-05

**Authors:** Dina Medina-Vera, Daniela Enache, Simone Tambaro, Ethar Abuhashish, Cristina Rosell-Valle, Bengt Winblad, Fernando Rodríguez de Fonseca, Erika Bereczki, Per Nilsson

**Affiliations:** Instituto de Investigación Biomédica de Málaga-IBIMA, Unidad de Gestión Clínica de Salud Mental, Hospital Regional Universitario de Málaga, Málaga 29010, Spain; Facultad de Ciencias, Universidad de Málaga, Málaga 29010, Spain; Facultad de Medicina, Universidad de Málaga, Málaga 29010, Spain; Department of Neurobiology, Care Sciences and Society, Center for Alzheimer Research, Division of Neurogeriatrics, Karolinska Institutet, 17164 Solna, Sweden; Department of Neurobiology, Care Sciences and Society, Center for Alzheimer Research, Division of Neurogeriatrics, Karolinska Institutet, 17164 Solna, Sweden; Department of Neurobiology, Care Sciences and Society, Center for Alzheimer Research, Division of Neurogeriatrics, Karolinska Institutet, 17164 Solna, Sweden; Instituto de Investigación Biomédica de Málaga-IBIMA, Unidad de Gestión Clínica de Salud Mental, Hospital Regional Universitario de Málaga, Málaga 29010, Spain; Department of Neurobiology, Care Sciences and Society, Center for Alzheimer Research, Division of Neurogeriatrics, Karolinska Institutet, 17164 Solna, Sweden; Theme Inflammation and Aging, Karolinska University Hospital, 17164 Solna, Sweden; Instituto de Investigación Biomédica de Málaga-IBIMA, Unidad de Gestión Clínica de Salud Mental, Hospital Regional Universitario de Málaga, Málaga 29010, Spain; Department of Neurobiology, Care Sciences and Society, Center for Alzheimer Research, Division of Neurogeriatrics, Karolinska Institutet, 17164 Solna, Sweden; Department of Microbiology, Tumor and Cell Biology & National Pandemic Center, Karolinska Institutet, 17177 Solna, Sweden; Department of Neurobiology, Care Sciences and Society, Center for Alzheimer Research, Division of Neurogeriatrics, Karolinska Institutet, 17164 Solna, Sweden

**Keywords:** synaptic proteins, ZnT3, GluA3, Alzheimer’s disease, *App* knock-in mice

## Abstract

Synaptic dysfunction is an early event in Alzheimer’s disease. Post-mortem studies suggest that alterations in synaptic proteins are associated with cognitive decline in Alzheimer’s disease. We measured the concentration of three synaptic proteins, zinc transporter protein 3, dynamin1 and AMPA glutamate receptor 3 in cerebrospinal fluid of subjects with mild cognitive impairment (*n* = 18) and Alzheimer’s disease (*n* = 18) and compared the levels to cognitively and neurologically healthy controls (*n* = 18) by using ELISA assay. In addition, we aimed to assess the translational potential of these synaptic proteins in two established amyloid precursor protein knock-in Alzheimer’s disease mouse models by assessing the cerebrospinal fluid, hippocampal and cortical synaptic protein concentrations. Using ELISA, we measured in parallel these three proteins in cerebrospinal fluid and/or brain of 12- and 24-month-old *App^NL-F^* and *App^NL-G-F^* knock-in mice and *App^Wt^* control mice. The regional distribution and expression of these proteins were explored upon aging of the *App* knock-in models by quantitative immunofluorescence microscopy. Notably, we found a significant increase in concentrations of zinc transporter protein 3 and AMPA glutamate receptor 3 in cerebrospinal fluid of both patient groups compared with cognitively healthy controls. Dynamin1 concentration was significantly higher in Alzheimer’s disease patients. Remarkably, patients with mild cognitive impairment who converted to Alzheimer’s disease (*n* = 7) within 2 years exhibited elevated baseline cerebrospinal fluid zinc transporter protein 3 concentrations compared with mild cognitive impairment patients who did not convert (*n* = 11). Interestingly, similar to the alterations in Alzheimer’s disease subjects, cerebrospinal fluid AMPA glutamate receptor 3 concentration was significantly higher in *App^NL-G-F^* knock-in mice when compared with wild-type controls. Furthermore, we have detected age and brain regional specific changes of the three synaptic proteins in the hippocampus and prefrontal cortex of both *App^NL-F^* and *App^NL-G-F^* knock-in mice. Notably, all the three cerebrospinal fluid synaptic protein concentrations correlated negatively with concentrations in hippocampal lysates. The elevated zinc transporter protein 3 concentrations in the cerebrospinal fluid of converter versus non-converter mild cognitive impairment patients suggests a prospective role of zinc transporter 3 in differentiating dementia patients of the biological continuum of Alzheimer’s disease. The increased cerebrospinal fluid concentrations of synaptic proteins in both patient groups, potentially reflecting synaptic alterations in the brain, were similarly observed in the amyloid precursor protein knock-in mouse models highlighting the translational potential of these proteins as markers for synaptic alterations. These synaptic markers could potentially help reduce the current disparities between human and animal model-based studies aiding the translation of preclinical discoveries of pathophysiological changes into clinical research.

## Teaser

This paper reports significant alterations of cerebrospinal fluid synaptic proteins throughout the biological continuum of Alzheimer`s disease. The high translatability of this synaptic profile from Alzheimer animal models to patients is essential to envisage the outcome of a treatment more accurately in patients according to Medina-Vera et al.

## Graphical Abstract

**Graphical abstract:**
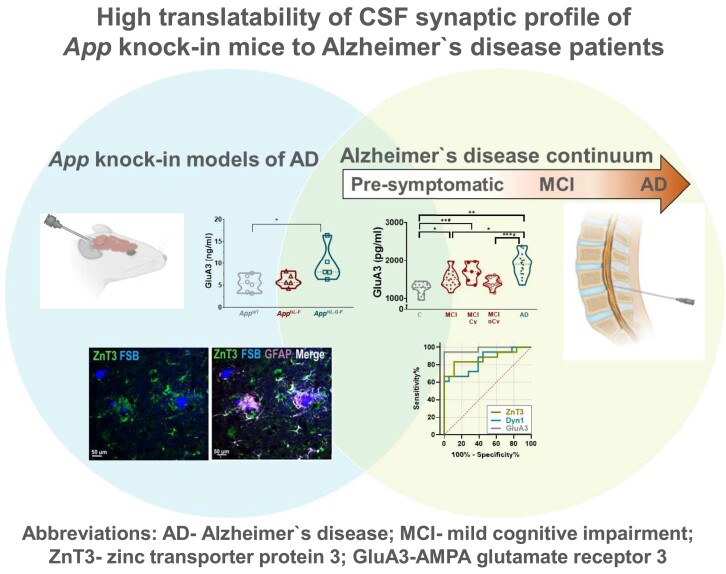

## Introduction

Alzheimer’s disease is the most common form of age-related neurodegenerative dementia with well-defined hallmarks: extracellular insoluble plaques composed of amyloid beta (Aβ) peptide, intracellular neurofibrillary tangles, glial inflammatory reaction and synaptic loss.^1^ The neurodegeneration in Alzheimer’s disease is characterized by both neuronal loss and synaptic degeneration and subsequent failure resulting in cognitive decline, behavioural and psychological symptoms and functional impairment.^2^ Diagnosing Alzheimer’s disease early has long been a challenge. By the time Alzheimer’s disease is clinically diagnosed, neuronal loss and neuropathologic lesions have already occurred in many brain regions.^3^

It is widely accepted that cognition declines through a biological continuum with a major part of the disease starting before the onset of neurocognitive symptoms encompassing stages from subjective cognitive decline (SCD), mild cognitive impairment (MCI) and Alzheimer’s disease dementia.^4,5^ MCI is defined both in clinic and research as a prodromal state of Alzheimer’s disease.^6^ While in the early preclinical stages of SCD no cognitive or pathological alterations are detected during neuropsychological assessment, in MCI early signs of memory and learning impairment, especially regarding the episodic memory, but also in domains of executive function, language and visual abilities are becoming evident^7^ which are further exacerbated in Alzheimer’s disease.^8^ The risk for conversion from MCI to Alzheimer’s disease dementia, has been estimated to be around 30% with annual conversion rates between 5 and 10%, depending on follow-up strategy and study design.^9^

For the past decade, researchers have been trying to identify high accuracy progression and differentiation Alzheimer’s disease markers. Currently, biomarkers, specifically CSF markers, can be used primarily as a research tool and optionally by physicians to identify at-risk patients for developing Alzheimer’s disease, with some patient stratification strategies considering their progression recently issued.^10^ The amyloid, tau and neurodegeneration (A/T/N) classification framework has paved the way for better patient stratification with increased risk profiling,^11,12^ however, we are still lacking high accuracy progression and differentiating markers for Alzheimer’s disease and other dementias. Various synaptic proteins, based on their role in synaptic transmission machinery, have been assessed previously in Alzheimer’s disease and other dementias. Some of the most promising, including neurogranin, neurofilament light chain and SV2A are being characterized in several patient cohorts monitoring their biomarker capacity.^13–17^ This promising progress in the field is especially encouraging and could boost drug development and aid devising clinical trials if such observational data is incorporated into new intervention designs.

Synaptic disturbance in Alzheimer’s disease is thought to be an early event with mild synaptic changes occurring already in patients with SCD. In neurodegenerative disorders, alterations in synaptic proteins are an essential predictive molecular fingerprint, and they could be a potential target for early disease intervention. Synaptic dysfunction is among the best correlate with cognitive decline in dementia and is thought to be an early common biological mechanism in neurodegenerative diseases such as Alzheimer’s disease.^18^ Post-mortem and recent CSF studies of dementia patients suggest that zinc transporter protein 3 (ZnT3), dynamin1 (Dyn1) and AMPA glutamate receptor 3 (GluA3) are associated with cognitive decline in Alzheimer’s disease.^18–21^ ZnT3 is located on the membrane of synaptic vesicles to transport zinc ions into synaptic vesicles.^22,23^ ZnT3 has been associated with Aβ plaque deposition^24^ and has been implicated in age-related cognitive decline.^25^ Dyn1 is a synaptic protein which plays a key role in endocytosis during synaptic transmission.^20^ Dyn1 is also involved in the modulation and formation of memory.^26^ GluA3, encoded by glutamate ionotropic receptor AMPA type subunit 3, is essential in glutamate excitatory signalling pathway during synaptic transmission.^27^ GluA3 has also been linked to Aß-induced effects on synapses leading to memory impairment.^28^

Common translational research efforts to deepen our understanding of the disease mechanism as well as testing novel therapeutic strategies require preclinical animal models that can accurately recapitulate complex human conditions. While transgenic mice overexpressing human amyloid precursor protein (App) with various mutations have yielded a lot of our current understanding of Alzheimer’s disease, these mice often develop artificial phenotypes related to the unphysiological overexpression of APP levels and its proteolytic fragments.^29^ To overcome this, we used two *App* knock-in mouse models previously generated as ‘models of preclinical Alzheimer’s disease’:^30^ the *App^NL-F^* and the *App^NL-G-F^* mice. These knock-in models exhibit physiological *App* expression leading to endogenous APP levels. The *App^NL-F^* knock-in mice harbour a humanized Aβ region, the Swedish ‘*NL*’ and the Beyreuther/Iberian ‘*F*’ mutations. These two mutations induce high Aβ_42_ levels and therefore high Aβ toxicity, as well as increase the ratio of Aβ_42_/Aβ_40_. The *App^NL-F^* knock-in mice recapitulate much of the Aβ related pathology observed in patients, developing initial deposition of Aβ plaques at six months and progressive cognitive impairments from 18 months of age.^29,30^ The *App^NL-G-F^* knock-in mice additionally carries the Arctic mutation ‘*G*’ which enhances Aβ oligomerization.^31^ The synaptotoxic effect of Aβ_42_ peptides is a significant constituent of the pathogenic process in Alzheimer’s disease.^32^ This effect is recapitulated in the *App* knock-in mice with accumulation of extracellular Aβ_42_ inducing mushroom like spine loss in the hippocampal neurons further contributing to cognitive impairment.^33^ The *App^NL-G-F^* knock-in mice start developing Aβ plaques from two months and early memory deficits from six months of age resulting in synaptic degeneration.^29,34^

In this study, we have compared the extent of synaptic changes as reflected by the concentrations of three synaptic proteins (ZnT3, Dyn1, GluA3), previously found to be associated with cognitive impairment and Alzheimer’s disease pathology.^18,35^ We have assessed their concentrations in CSF and brains of Alzheimer’s disease animal models as well as in CSF of Alzheimer’s disease, MCI and cognitively and neurologically healthy controls. We hypothesized that Alzheimer’s disease and MCI patients exhibit higher CSF concentrations of ZnT3, Dyn1 or GluA3 compared with healthy controls. As translatable synaptic biomarkers could potentially help reduce disparities between human and animal model-based studies facilitating the translation of preclinical discoveries of pathophysiological changes into clinical research, we also explored the synaptic alterations in two *App* knock-in mouse models. We therefore have assessed the CSF, hippocampal and cortical concentrations of ZnT3, Dyn1, GluA3 within the same animal in a disease severity, age-dependent and brain regional dependent manner.

## Materials and methods

### Participants

In total, 54 patients (18 cognitively and neurologically healthy control subjects, 18 MCI and 18 Alzheimer’s disease) recruited from Karolinska University Hospital, Huddinge, Sweden were included in the study. MCI and Alzheimer’s disease patients were referred to the Memory clinic between 2010 and 2013. MCI patients were followed for at least 1 year (mean follow-up was 2.1 years), with follow-up data being collected from patient files by November 2013. Seven out of 18 MCI patients have converted during this follow-up time to Alzheimer’s disease. All the participants gave their written consent prior to lumbar puncture procedure for the use of their clinical data and CSF for research purposes. The study was approved by the regional committee for ethics in medical research in Stockholm (DNR 2012/920-31/4) and was performed in compliance with the STROBE guidelines.^36^ Healthy control subjects were diagnosed with benign neurological diagnoses, such as tension headaches with no evidence of dementia, no cognitive complaints or other brain diseases. Inclusion criteria for Alzheimer’s disease and MCI patients consisted of being diagnosed with Alzheimer’s disease, or MCI and having measured CSF biomarkers T-tau, p-tau and Aβ_42_. Exclusion criteria were being diagnosed with other neurological conditions affecting CSF measurements (e.g. brain tumours, multiple sclerosis, hydrocephalus, cerebrovascular disease). Participants (except the cognitively and neurologically healthy control subjects for whom neurological conditions were out ruled) underwent a medical examination that included a cognitive screening using the Mini-Mental State Examination (MMSE), a standardized neuropsychological examination, routine blood chemistry, CSF and brain imaging scans. Alzheimer’s disease and MCI diagnoses were set according to the core clinical criteria of the National Institute on Aging—Alzheimer’s Association Framework on Alzheimer’s disease^5^ and according to the revised clinical criteria of Winblad *et al*.^37^

### Cerebrospinal fluid sampling

CSF samples were taken on the first visit to the memory clinic. Samples were obtained by lumbar puncture between the L3/L4 or L4/L5 intervertebral space using a 25-gauge needle, in the morning hours (within a 3 h interval) according to a standardized protocol. Samples were collected into polypropylene-tubes and subsequently centrifuged at 1300–1800 × *g* at 4°C for 10 min before being stored in aliquots of 100 μl at −80°C as previously described.^38^ CSF concentrations of Aβ42, total tau (T-tau), and tau phosphorylated at threonine 181 (p-tau) were measured by commercially available sandwich ELISAs (Innogenetics, Ghent, Belgium).

### Animals and ethics statement

We used wild-type (*App^wt^*), and *App* knock-in *App^NL-F^* and *App^NL-G-F^* mice harbouring Swedish^39^ and Beyreuther/Iberian^40^ mutations with and without the Arctic^31^ mutation in the *App* gene. All experiments included both females and males at 12- and 24-months of age and were performed in compliance with the ARRIVE guidelines^41^ and in concordance with the European Communities Council Directives 2010/63/EU, Regulation (EC) No. 86/609/ECC (24 November 1986). A total of 30 mice across the genotypes and ages (Table 1) were used in this study reflecting our efforts to reduce the number of animals used. The animal experiments were performed under permit ID407 obtained from the Linköping animal ethic board.

**Table 1:** Demographics and clinical characteristics of patient cohort and number of animals included in the study

Patient cohort	C	MCI	MCI-nCv	MCI-Cv	Alzheimer’s disease	*P*-value
Age (y)	65.2 ± 7.8	65.5 ± 8.2	63.2 ± 6.4	69.3 ± 9.9	65.2 ± 7.8	ns
*n*	18	18	11	7	18	ns
Gender % M/F	44%	39%	45%	28%	39%	ns
YofEd	NA	11.4 ± 2.4	11.8 ± 2.5	10.7 ± 2.2	11.6 ± 3.7	ns
MMSE	NA	27.2 ± 1.6	27 ± 1.7	27.4 ± 1.7	21.2 ± 2.5	<0.0001^*¤#^
Aβ_42_ pg/ml	NA	718.3 ± 248.9	829.1 ± 203.9	544.1 ± 219.9	661.7 ± 194.8	**0.013^¤^; 0.036^#^**
T-tau pg/ml	NA	423.7 ± 231.9	341.3 ± 160.9	553.3 ± 277.7	743.5 ± 367.0	0.004* ; 0.002^#^
p-tau pg/ml	NA	71.4 ± 26.5	61.7 ± 24.6	85.5 ± 23.9	93.5 ± 35.8	0.039* ; 0.015^#^
ZnT3 pg/ml	1357 ± 145	1662 ± 325	1455 ± 161	1987 ± 233	1788 ± 341	**0.0347^§^;<0.0001^ʘ^; 0.002^¤^; 0.0007°; 0.0039^#^**
Dyn1 pg/ml	494 ± 38.3	522 ± 50.6	509 ± 54.4	542 ± 38.9	578 ± 78.6	**0.016°**
GluA3 pg/ml	1259 ± 152	1512 ± 223	1402 ± 138	1685 ± 229	1881 ± 306	0.0394^§^; 0.0005^ʘ^;<0.0001°; 0.0289*;<0.0001^#^

Animal cohort	WT (ELISA/IHC)		*APP^NL-F^*(ELISA/IHC)		*APP^NL-G-F^*(ELISA/IHC)	
Age	12 m	24 m	12 m	24 m	12 m	24 m
Gender % M/F	25%	67%	75%	20%	25%	71%
*n*	4/4	6/4	4/4	5/4	4/4	4/5

Data are represented as mean ± SD. The differences between the groups were assessed using Kruskal–Wallis`s test followed by the Dunn multiple comparisons test. Statistical significance is highlighted in bold and is considered significant at *p* < 0.05. § denotes comparison between C versus MCI, * between MCI versus Alzheimer’s disease cases, ° between C versus Alzheimer’s disease, ʘ between C and MCI CV, ¤ between MCI-nCv and MCI-Cv and # between MCI nCv versus Alzheimer’s disease cases.

APP, amyloid precursor protein; C, control; Cv, converter; nCv, non-converter; CSF, cerebrospinal fluid; F, female; MCI, mild cognitive impairment; M, male; MMSE, mini mental state examination; NA, not available; T, total; p, phosphorylated; WT, *App^wt^*; YofEd, years of education.

### Mouse cerebrospinal fluid and brain samples

Mouse CSF from 24-month-old animals was sampled from anaesthetized mice using 2% isoflurane kept on a thermopad to prevent hypothermia. A scission was performed mid-skull, and muscles were dissected by blunt end dissection to expose the dura mater, which was carefully cleaned from blood with a swab wetted in phosphate buffer saline (PBS). The dura mater was penetrated with a needle gauge 27 and about 10 μl CSF was subsequently collected in a glass capillary, assessed for blood contamination, and directly transferred to low affinity Eppendorf tubes and snap frozen in liquid nitrogen. Mice were perfused with PBS through cardiac perfusion, and brains were dissected thereafter.

Preparation of mouse brain tissue from 12- and 24-month-old animals for ELISA analyses was performed according to the following. Briefly, frozen tissue was homogenized in ice-cold RIPA buffer (Thermo Fisher Scientific) supplemented with protease inhibitor cocktail tablets (Roche, 1 tablet per 50 ml of buffer). The buffer was used at a ratio of 2 ml to every 100 mg of tissue, and homogenization was performed using an IKA Ultra-Turrax mechanical probe (IKA Werke, Germany) until the liquid appeared homogeneous. After a brief centrifugation, protein concentration of each sample was measured by using BCA Protein Assay Kit (Pierce, Thermo Fisher Scientific). Samples for ELISA measurements were further diluted to 0.5 μg/μl total protein in PBS.

### Sandwich enzyme-linked immunosorbent assays

Commercial sandwich ELISA kits for ZnT3, GluA3 and Dyn1 were purchased from Antibodies online (human and mouse ZnT3 and Dyn1) or Mybiosource (mouse and human GluA3). Assay procedures were performed according to the manufacturer’s protocol. A 100 µl of GluA3, ZnT3 or Dyn 1 standards or samples were applied to the ELISA plates and incubated with the corresponding antibody for 2 h at 37°C, followed by three washing steps. Incubation with detection reagent was followed by three washing steps and the addition of chromogen solution. After stopping the reaction, absorbance was measured immediately at 450 nm on a SpectraMax Plus384 microplate reader (Molecular Devices). The sigmoidal standard curve was evaluated with non-linear four-parameter fit using SoftMax Pro 5.2 software and sample amounts were obtained using the fitted standard curve. Standards and samples were measured in duplicates and standards were diluted so that the sample absorbance values would fall near 50% binding (the linear range) of the standard curve. Concentrations were calculated after the mean blank value had been subtracted.

### Histological and immunofluorescence analysis

After perfusion with PBS under anaesthetization by isoflurane, hemibrains were fixed in 10% formalin solution (Merck Millipore, Cat. no: HT501128), dehydrated and paraffin embedded. Coronal sections of 5 μm thickness were de-paraffinized by washing in xylene and re-hydrated in decreasing concentrations of ethanol (from 99 to 70%). After antigen retrieval, and incubation in TNB blocking buffer (TSA Blocking Reagent Cat#FP1020, Akoya Biosciences), sections were incubated at 4°C O/N with primary antibodies: polyclonal rabbit anti-ZnT3 (1:250) (Cat#197 003, Synaptic Systems), polyclonal rabbit anti-Dyn1 (1:200) (Cat#PA1-660, Invitrogen), polyclonal rabbit anti-GluA3 (1:200) (Cat#ab232887, Abcam). After thorough washing in PBST, sections were incubated with biotinylated goat anti-rabbit secondary antibody (Cat#BA-1000, Vector Laboratories Inc.) (1:200) at RT for 2 h and after a washing step, sections were incubated with Streptavidin-HRP reagent (TSA Fluorescein System Kit, Cat#NEL701A001KT, Akoya Biosciences) (1:100) for 30 min. Thereafter, slides were washed in PBST and incubated with fluorophore Tyramide (TSA Fluorescein System Kit, Cat#NEL701A001KT, Akoya Biosciences) (1:50) in Amplification Reagent (TSA Fluorescein System Kit, Cat#NEL701A001KT, Akoya Biosciences) for 10 min. For Aβ plaque detection, slides were further incubated in 1-fluoro-2,5-bis(3-carboxy-4-hydroxystyryl) benzene (FSB) (Cat#344101-5MG, Millipore) (1:1000) at RT for 30 min. Lastly, slides were washed in PBST and mounted with Vectashield HardSet Antifade Mounting Medium with DAPI (Cat#H-1500, Vector Laboratories) or PermaFluor water soluble mounting media (Cat#TA-030-FM, Thermo Scientific) if FSB staining was done.

For double staining, a second primary antibody for microglia (rabbit anti-Iba1, Cat#CAK1997, Wako) or astrocytes (rabbit anti-GFAP, Cat#Z0334, DAKO), (1:100) was applied after incubation with fluorophore Tyramide for 10 min and washing the slides with PBST, and incubated at +4°C O/N. After thorough washing, the sections were incubated with Alexa Fluor 546 conjugated goat anti-rabbit secondary antibody (Thermo Fisher, Cat#A-11035) (1:500) at RT for 2 h followed by washing and mounting steps as previously described. Negative control slides were used for every staining. All batches of immunostaining contained slides from all genotypes. All analyses were performed blinded to the identity of the samples.

### Microscopy and fluorescence intensity quantification

Images were acquired with Nikon Eclipse E800 with a Plan-Apochromate 2×, 4×, 10×, and 20× objectives. NIS-Elements D software, version 4.30.00, was used for image processing. We used ImageJ software^42^ for fluorescence intensity quantification. All pictures were taken with the same excitation light intensity, exposure time and analogue gain. The images were then binarized to 8-bit black, and a fixed intensity threshold was applied for each immunostaining. We outlined the region of interest with the ROI tool and Set Area (resulting in same size), Integrated Density and Mean Grey Value as the desired parameters to analyse. Four to six mice per group and two sections per mouse were used. For better image quality (not included in the quantification), selected images were taken also with Zeiss LSM800 using ZEN software, with oil immersion with a 63 × objective.

### Statistical analyses

Analyses were performed using the IBM SPSS statistics software (version 25) and GraphPad Prism (version 9). The Shapiro–Wilk test was used to assess normal distribution of data. Patients across the diagnoses were selected to have matching age and gender, therefore no further corrections for these factors were applied. Kruskal–Wallis’s test was used for comparisons among the different patient groups, as well as between the animal groups, followed by Dunn’s multiple comparisons test. A separate Kruskal–Wallis’s analysis, omitting the MCI group was performed when MCI Cv and MCI nCv groups were compared with healthy controls and Alzheimer’s disease groups. Receiver operating characteristic (ROC) curve analyses were performed for each synaptic protein in order to assess their diagnostic value and calculate the area under the curve, sensitivity and specificity as well as 95% confidence intervals. The analysis of two single groups when a third comparison group was not available [analysis of the Piriform cortex (PC) in 12-month-old animals] was performed using Student’s unpaired *t*-test. Correlation analyses between synaptic protein concentrations in murine CSF and brain regions were assessed by Pearson`s correlations. All ELISA measurement data are expressed as mean ± standard deviation (SD) and all image quantification data is presented as the mean ± standard error of the mean (SEM) or as stated in the figure legends, and *P*-values less than 0.05 were considered statistically significant.

### Data availability

The data supporting the findings in this article are available in Supplementary material, with further data available from the corresponding author upon reasonable request.

## Results

### Demographic and clinical characteristics of patient cohort

The key demographic and clinical cohort characteristics are shown in Table 1. As the patient cohorts were selected to be similar in age and gender, there were no significant differences regarding these two factors between total MCI, Alzheimer’s disease and control groups. While there were no significant differences in the years of education, the MCI-Cv group had a tendency for lower education, nevertheless disease specific markers such as MMSE scores, Aβ_42_, T-tau, p-tau were significantly differing between MCI and Alzheimer’s disease groups (Table 1).

### Increased cerebrospinal fluid levels of zinc transporter 3, dynamin1 and AMPA glutamate receptor 3 levels in Alzheimer’s disease and mild cognitive impairment patients

**Figure 1:**
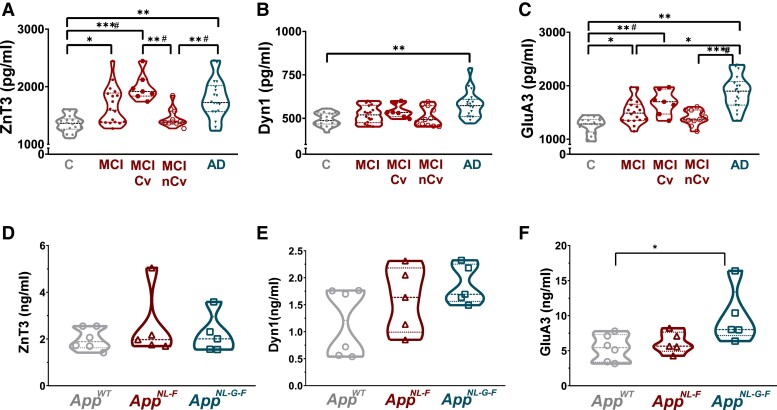
**Increased CSF concentrations of synaptic proteins in dementia patients (A–C) and *App* knock-in mice (D–F).** Violin plots represent synaptic protein concentrations measured by ELISA showing median, quartiles and individual values (Kruskal–Wallis’s test followed by Dunn’s multiple comparison) (**A–C**) All three synaptic proteins show significantly elevated levels in Alzheimer’s disease compared with control cases. Additionally, ZnT3 (**A**) and GluA3 (**C**) concentrations are elevated in MCI patients. When MCI group was further stratified based on the available clinical follow-up data, MCI Cv group show elevated ZnT3 concentrations in comparison to MCI NCv group. Also, MCI nCv concentrations are significantly lower than Alzheimer’s disease cases. (**D–F**) CSF concentrations of GluA3 (**F**) were significantly increased in *App^NL-G-F^* knock-in mice compared with *App^wt^* mice. All values are shown as the mean ± SD. In all cases * denotes significance with *P* ≤ 0.05, **P ≤ 0.01, *** *P* ≤ 0.001 level; # denotes comparison including C, MCI Cv, MCI nCv and Alzheimer’s disease cases only. Abbreviations: C, control; Cv, converter; CSF, cerebrospinal fluid; MCI mild cognitive impairment; nCv, non-converter.

The concentrations of ZnT3, Dyn1 and GluA3 were significantly elevated in the CSF of Alzheimer’s disease patients compared with the age and gender matched control group (*P* = 0.003 for ZnT3, *P* = 0.007 for Dyn1 and *P* < 0.001 for GluA3, Table 1, Fig. 1A–C). Notably, concentrations of both ZnT3 (*P* = 0.011) and GluA3 (*P* = 0.0194) were found to be significantly increased in the MCI group when compared with control subjects (Table 1, Fig. 1A and C). When assessing the differences between MCI and Alzheimer’s disease groups, we further found that GluA3 (*P* = 0.015) concentrations were significantly higher in Alzheimer’s disease cases (Fig. 1C). As MCI patient cohorts are usually heterogeneous, containing patients at different stages of the neurodegenerative process potentially hampering the prognostic estimation, we further stratified them based on the available clinical follow-up data (note, no follow-up CSF sample was available to reassess synaptic levels) into MCI-converter (MCI-Cv, seven patients) and MCI non-converter (MCI-nCv 11 patients) groups. Even though this stratification reduced the number of participants, it revealed that the MCI-Cv group exhibited significantly higher ZnT3 CSF concentrations when compared with the MCI-nCv group (*P* = 0.0002). Interestingly, both ZnT3 and GluA3 concentrations of MCI-nCv were significantly lower than the concentrations measured in Alzheimer’s disease group (*P* = 0.004, and *P* < 0.0001, respectively, Table 1).

### Diagnostic utility of synaptic proteins in cerebrospinal fluid of mild cognitive impairment and Alzheimer’s disease patients

Importantly, we found that all the three synaptic proteins exhibited excellent diagnostic utility in differentiating control cases from MCI or Alzheimer’s disease (Table 2, Supplementary Fig. 1). In the case of Alzheimer’s disease, the highest area under the curve (AUC) was observed for GluA3 equal to 0.98 (sensitivity 94%, specificity 100%), 0.85 AUC for Dyn1 (sensitivity of 67% and specificity of 94%) and 0.88 AUC (sensitivity of 83% and specificity of 89%) in the case of ZnT3 (Table 2, Supplementary Fig. 1). Notably, the diagnostic utility of GluA3 was high with 0.95 AUC in differentiating MCI Cv group compared with control cases with 88% sensitivity and 100% specificity (Table 2, Supplementary Fig. 1).

**Table 2:** Diagnostic utility of ZnT3, GluA3 and dynamin 1 in CSF of patients with various clinical dementias

	Models	AUC	Sensitivity (95% CI)	Specificity (95% CI)
ZnT3	MCI	**0.78**	**50% (29.1–70.9)**	**100% (82.4–100)**
	MCI- nCv	**0.86**	**64% (35.4–84.8)**	**100% (82.4–100)**
	MCI-Cv	0.67	86% (48.7–99.3)	50% (29.1–70.9)
	Alzheimer’s disease	**0.88**	**83% (60.8–94.2)**	**89% (67.2–98.1)**
Dyn1	MCI	0.66	72% (49.1–87.5)	56% (33.7–75.4)
	MCI-nCv	0.57	36% (15.2–64.6)	94% (74.2–99.7)
	MCI-Cv	**0.8**	**100% (64.6–100)**	**56% (33.7–75.4)**
	Alzheimer’s disease	**0.85**	**67% (43.8–83.7)**	**94% (74.2–99.7)**
GLuA3	MCI	**0.82**	**89% (67.2–98.1)**	**61% (38.6–79.7)**
	MCI-nCv	**0.75**	**82% (52.3–96.7)**	**61% (38.6–79.7)**
	MCI-Cv	**0.94**	**86% (48.7–99.3)**	**100% (82.4–100)**
	Alzheimer’s disease	**0.98**	**94% (74.3–99.7)**	**100% (82.4–100)**

Results from ROC curve analyses are visualized in panel **A-D** and summarized with area under curve (AUC), sensitivity and specificity of synaptic proteins expressed as % with 95% confidence intervals (CI) in parenthesis. Models that were significant (*P* < 0.05) are presented in bold.

MCI, mild cognitive decline; Cv, converters; nCv, non-converters.

### Elevated cerebrospinal fluid levels of synaptic proteins in *App* knock-in mice

In a translational approach, we investigated the changes of the three synaptic proteins upon progression of the pathologies in two *App* knock-in mice, *App^NL-F^* and *App^NL-G-F^*, exhibiting robust Aβ pathology, neuroinflammation and cognitive impairments, by assessing the concentrations of ZnT3, Dyn1 and GluA3 in 24-month-old *App^NL-F^* and *App^NL-G-F^* mice compared with age matched *App^wt^* mice by ELISA. Four to six mice per group were used throughout the analysis (Table 1). Notably, similarly to MCI and Alzheimer’s disease patients, CSF concentrations of GluA3 were significantly higher in the 24-month-old *App^NL-G-F^* knock-in mice when compared with *App^wt^* mice (Fig. 1F). ZnT3 and Dyn1 concentrations in CSF did not change significantly though there was a trend towards a slight increase of Dyn1 levels in the *App* knock-in models (Fig. 1D and E, *P* = 0.06).

### Distinct expression pattern of zinc transporter 3, dynamin1 and AMPA glutamate receptor 3 in *App* knock-in mice

**Figure 2:**
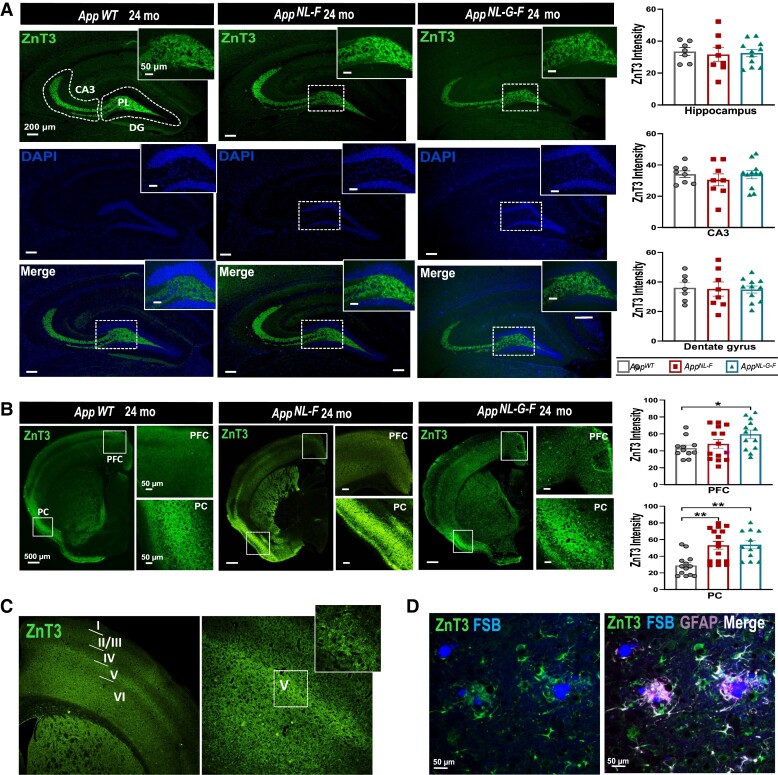
**Expression and localization of ZnT3 in 24-month-old *App*^wt^, *App^NL-F^*, *App^NL-G-F^* mice.** (**A**) Immunofluorescence images show high expression of ZnT3 in the CA3 and polymorphic layer (PL) of the dentate gyrus (DG) region of the hippocampus, revealing no significant genotype dependent changes. (**B**) Increased cortical ZnT3 immunoreactivity has been found in the PFC and PC of 24-month-old *App^NL-F^* and *App^NL-G-F^*. (**C**) Strong ZnT3 immunoreactivity in the internal pyramidal cell layer (V) of the PFC was observed, whereas ZnT3 expression was absent in the internal granular layer (IV). (**D**) Localization of ZnT3 in astrocytes in *App^NL-G-F^* mouse brain was detected in the 24-month-old *App^NL-G-F^* mice. Scale bars represent 500 μm, 200 μm and 50 μm (as indicated). Data represent mean ± SEM. (*n* = 4–6 mice per group, and two sections per mouse were used for quantification). In the case of *App*^wt^, *App^NL-F^* three male and one female mouse were included, while for *App^NL-G-F^* four male and two female mice were analysed. Kruskal–Wallis`s test followed by Dunn’s multiple comparison’s test has been used, * denotes *P* < 0.05, ** denotes *P* < 0.01 and *** denotes *P* < 0.001. Abbreviations: PFC, prefrontal cortex; PC, piriform cortex; FSB, 1-Fluoro-2,5-bis(3-carboxy-4-hydroxystyryl) benzene; GFAP, Glial fibrillary acidic protein.

**Figure 3:**
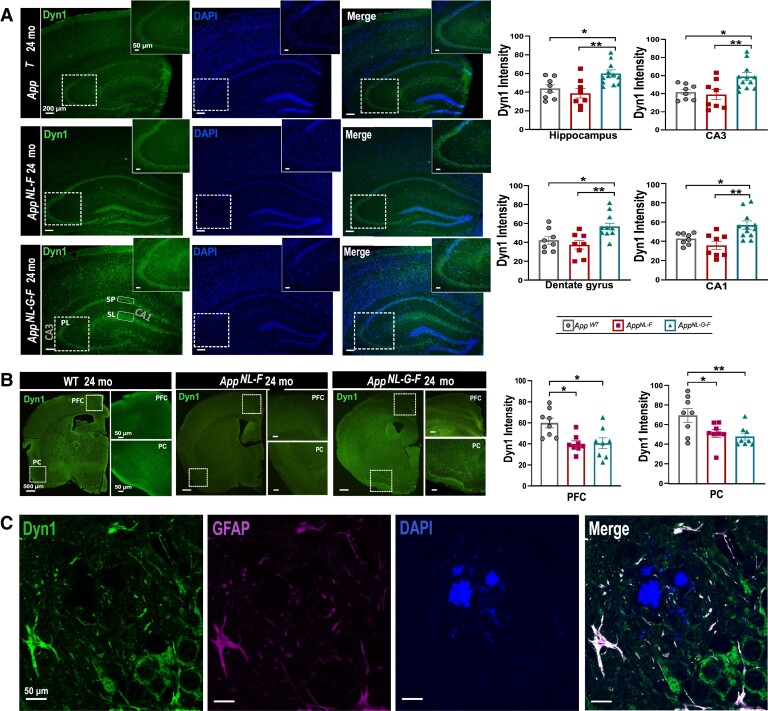
**Expression and localization of Dyn1 in 24-month-old *App* knock-in mice**. (**A**) Immunofluorescence images revealed increased Dyn1(green) immunoreactivity in the CA3, CA1 and dentate gyrus (DG) region of the hippocampus, more specifically in the stratum pyramidale (SP) and the stratum lacunosum (SL) of *App^NL-G-F^* mice. (**B**) Decreased Dyn1 immunoreactivity was observed in the prefrontal and piriform cortex of both *App* knock-in models. (**C**) Representative images showing marked astrocytosis and gliosis in *App^NL-G-F^* mouse brain around amyloid plaques (GFAP; FSB) and astrocytes. Scale bars represent 500 μm, 200 μm or 50 μm as indicated. Data represent mean ± SEM. (*n* = 4–6 mice per group, and two sections per mouse were used for quantification). In the case of *App*^wt^, *App^NL-F^* three male and one female mouse were included, while for *App^NL-G-F^* four male and two female mice were analysed. Kruskal–Wallis`s test followed by Dunn`s multiple comparison`s test has been used: (*) *P* < 0.05 and (**) *P* < 0.01. Abbreviations: PFC, prefrontal cortex; PC, piriform cortex; FSB, 1-Fluoro-2,5-bis(3-carboxy-4-hydroxystyryl) benzene; GFAP, glial fibrillary acidic protein.

**Figure 4:**
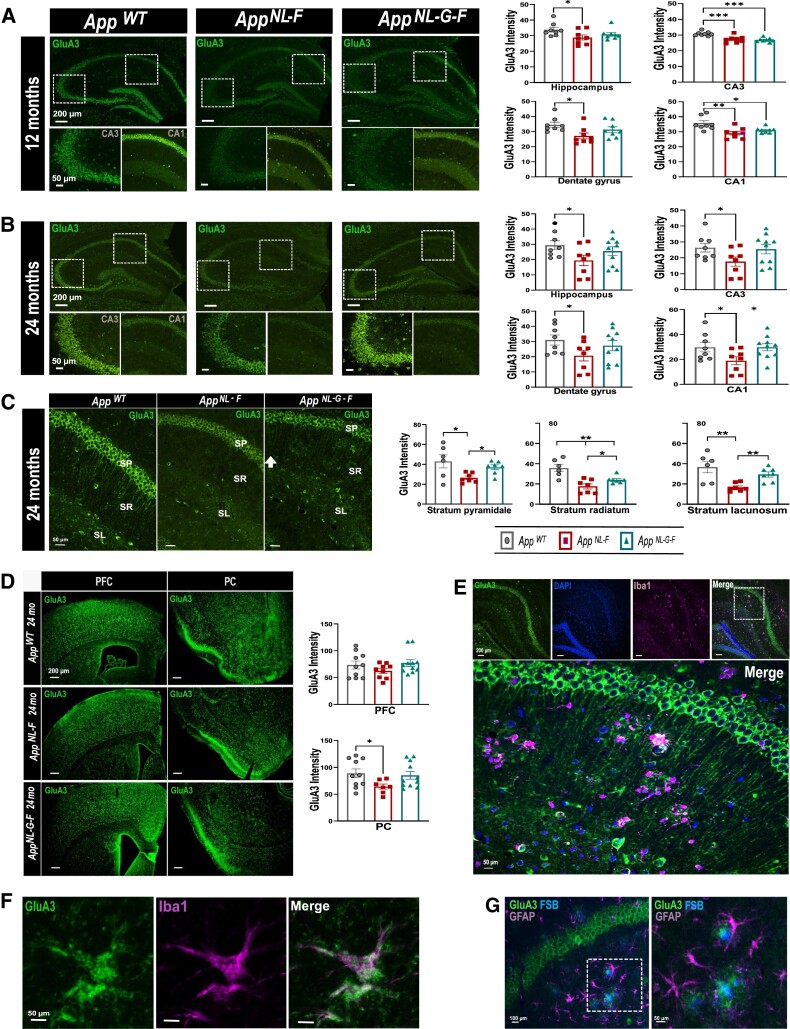
**Expression and localization of GluA3 in 12- and 24-month-old *App* knock-in mice.** (**A, B**) Brain sections from 12- and 24-month-old *App^wt^, App^NL-F^, App^NL-G-F^* mice show wide distribution of GluA3 in CA3, CA1 and dentate gyrus (DG) region of the hippocampus. Representative images show an overall decrease of GluA3 immunoreactivity throughout the hippocampal structures in both *App* knock-in models at 12 months of age and in 24-month-old *App^NL-F^* mice. (**C**) In the CA1 region, GluA3 immunofluorescence was predominantly located in the stratum piramidale (SP) with moderate immunofluorescence detected also in the stratum radiatum (SR) and stratum lacunosum (SL). Throughout the whole CA1 region, GluA3 immunoreactivity was decreased in 24-month-old *App* knock-in models when compared with *App^wt^* mice. (**D**) Decreased GluA3 immunoreactivity was observed in the piriform cortex (PC) of 24-month-old *App^NL-F^* mice. (**E, F**) Representative images of Iba1 and Glu3A reveal co-staining of microglia and GluA3 in *App^NL-G-F^* mouse brain with strong GluA3 immunoreactivity around FSB-positive Aβ plaques. (**G**) GFAP and GluA3 immunostaining reveals absence of GluA3 immunoreactivity in astrocytes. Scale bars represent 200 μm or 50 μm. Data represent mean ± SEM. (*n* = 4–6 mice per group, and a minimum of two sections per mouse were used for quantification). In the case of *App*^wt^, *App^NL-F^* three male and one female mouse were included, while for *App^NL-G-F^* four male and two female mice were analysed. Kruskal–Wallis`s test followed by Dunn`s multiple comparison`s test has been used, * denotes *P* < 0.05, ** denotes *P* < 0.01 and *** denotes *P* < 0.001.

Having found alterations in CSF of the *App* knock-in mice of the three synaptic proteins, we continued analysing the brain-regional distribution and immunoreactivity pattern of ZnT3, Dyn1 and GluA3 using immunofluorescence in 12- and 24-month-old *App* knock-in mice compared with age matched *App^wt^* mice to identify regions with altered levels that could explain the changes in CSF. Marked, yet distinct pattern of hippocampal and cortical immunostaining of these three proteins was noticed (Figs. 2–4, Supplementary Fig. 2A).

ZnT3 was highly expressed in the CA3 and the polymorphic layer (PL) of the dentate gyrus (DG) region of the hippocampus (Fig. 2A). While a strong ZnT3 immunoreactivity is seen in the hippocampal mossy fibre pathway, no apparent difference in staining intensity was found between the genotypes neither at 12 nor 24 months of age (Fig. 2A, Supplementary Fig. 2A and B). However, cortical ZnT3 analysis (Fig. 2B) revealed increased immunoreactivity in the PFC and the PC of 24-month-old *App* knock-in mice compared with *App^wt^* mice (Kruskal–Wallis test followed by Dunn’s multiple comparison test: *P* < 0.005 for PFC and *P* < 0.001 for PC). Interestingly, we have also noted higher immunoreactivity in layer V of the cortex, with low immunoreactivity in the internal granular layer IV (Fig. 2C). Double immunofluorescence staining was performed to assess the expression of ZnT3 in microglia, assessed by Iba1 and astrocytes assessed by GFAP staining. As previously shown, abundant immunoreactivity of activated microglia in *App^NL-F^* and *App^NL-G-F^* mice is present at 12 months of age and further enhanced at 24 months of age, (Supplementary Fig. 3A). However, no ZnT3 expression was observed in microglia cells (Supplementary Fig. 3B). Interestingly, in addition to neurons, ZnT3 was also present in astrocytes, especially around the FSB-positive Aß plaques with a noted accumulation of ZnT3 in astrocytes around these Aβ plaques (Fig. 2D).

We next examined Dyn1 immunoreactivity. Whereas no marked changes were observed in 12-month-old mice across the genotypes (Supplementary Fig. 2C), at 24 months of age, the hippocampal immunoreactivity of Dyn1 was significantly increased in the *App^NL-G-F^* mice (Fig. 3A). In the hippocampal CA1 region of the *App^NL-G-F^*, Dyn1 was highly expressed in the stratum pyramidale (SP) and the stratum lacunosum (SL) (Fig. 3A). In contrast, in the *App^wt^* and *App^NL-F^* mice, the expression was only marginal in the SP (Fig. 3A). In DG as well as in CA3, we also observed higher immunoreactivity of Dyn1 in the pyramidal cell layer (PL), especially in *App^NL-G-F^* mice (Fig. 3A, *P* < 0.005). In addition, we found a significant decrease of Dyn1 immunoreactivity in both the *App^NL-F^* and the *App^NL-G-F^* PFC and PC regions compared with those of the *App^wt^* mice (*P* < 0.005, Fig. 3B). Interestingly, Dyn1 is also present in astrocytes around the Aβ plaques (Fig. 3C), while no Dyn1 immunoreactivity was observed in microglia (Supplementary Fig. 3C).

GluA3 hippocampal immunoreactivity was decreased in both 12- and 24-month-old *App* knock-in mice when compared with the *App^wt^* control mice (Fig. 4A and B). In the CA1 region (Fig. 4C), the expression of GluA3 was predominantly located in the SP but was also detected in the axons of the stratum radiatum (SR) with decreased immunoreactivity in both the *App^NL-F^* and *App^NL-G-F^* mice (Fig. 4C, *P* < 0.005). Analysis of cortical GluA3 immunoreactivity (Fig. 4D), revealed a significant drop in the PC of the *App^NL-F^* mice at 24 months of age (*P* < 0.005). Additionally, we found strong GluA3 immunoreactivity around FSB-positive Aβ plaques most likely in microglia since GluA3 was not only expressed in activated (Fig. 4E), but also in resting microglia while being absent in astrocytes (Fig. 4E–G).

### Age-dependent synaptic alterations in the *App* knock-in mice

**Figure 5:**
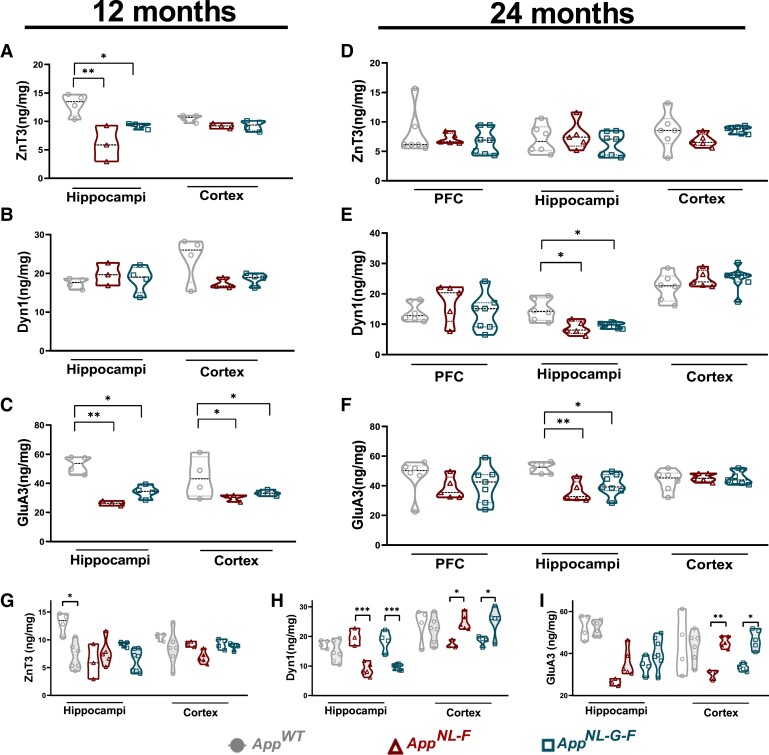
**Altered concentration of ZnT3, Dyn1 and GluA3 in the brains of *App* knock-in mice.** Violin plots represent synaptic protein concentrations measured by ELISA showing median, quartiles and individual values; differences between the groups were assessed by Kruskal–Wallis`s test followed by Dunn`s multiple comparison test. Overall hippocampal decrease in ZnT3 (**A**) and GluA3 (**C**) concentrations, with additional cortical drop in GluA3 concentrations were found in both 12-month-old *App* knock-in mice. (**B**) No major changes in Dyn1 concentrations were detected at 12 months. (**D**) Concentrations of ZnT3 in 24-month-old animals showed similar levels throughout all the genotypes. Reduction of both Dyn1 (**E**) and GluA3 (**F**) was observed in the hippocampi of both 24-month-old *App* knock-in models when compared with *App^wt^* mice. Age-related concentration changes in the 12- (transparent shape) and 24-month-old (filled shape) *App* knock-in mice (**G**, **H**, **I**). In all cases *denotes significance with *P ≤ 0.05 **P ≤ 0.01, *** *P* ≤ 0.001. Abbreviations: CSF, cerebrospinal fluid; PFC, prefrontal cortex.

The concentrations of the three synaptic proteins in the brains of the *App* knock-in mice were further assessed by ELISA revealing an overall concordance with immunofluorescence quantification results with occasional divergences. A significant drop of both ZnT3 (Fig. 5A) and GluA3 (Fig. 5C) was observed at 12 months of age in the hippocampi of both *App^NL-F^* and *App^NL-G-F^* mice when compared with *App^wt^* mice. A drop of GluA3 concentration was also observed in the cortex of both *App* knock-in mice (Fig. 5C). No changes were detected in Dyn1 concentration at this age (Fig. 5B). At 24 months of age, the concentration of ZnT3 protein was not changed significantly in either knock-in model compared with aged matched *App^wt^* mice (Fig. 5D). We observed a significant decrease in Dyn1 concentrations in hippocampal lysates of both *App^NL-F^* and *App^NL-G-F^* mice when compared with *App^wt^* mice, while no significant differences were observed in PFC or in cortex (Fig. 5E). Furthermore, similarly to the 12-month-old mice, there was a significant decrease in GluA3 concentrations in hippocampal lysates of both *App^NL-F^* and *App^NL-G-F^* knock-in mice when compared with *App^wt^* mice (Fig. 5F). No changes have been observed in the concentration of neither synaptic proteins analysed in prefrontal cortex nor in the cortex of the old *App* knock-in animals when compared with age matched *App^wt^* mice, further highlighting the conspicuous changes observed in hippocampus.

We have found an overall age-related decrease in the hippocampal concentration of Dyn1 in both 24-month-old *App* knock-in models, with a trend towards reduction in the *App^wt^* mice (Fig. 5G). Interestingly, no time-dependent changes of ZnT3 and GluA3 in hippocampus of either *App* knock-in models were observed (Fig. 5H and I), whereas decreased hippocampal ZnT3 concentrations were noted in the old *App^wt^* mice compared with the younger 12-month-old mice (Fig. 5G). In contrast, while the cortical concentration of both Dyn1 and GluA3 in *App^wt^* mice was unchanged between the two ages analysed, we have found elevated cortical Dyn1 and GluA3 protein concentrations in the 24-month-old *App^NL-F^* and *App^NL-G-F^* mice when compared with younger, 12-month-old *App* knock-in mice (Fig. 5H and I).

### Cerebrospinal fluid levels of zinc transporter 3, dynamin1 and AMPA glutamate receptor 3 correlates negatively with brain regional changes

**Figure 6:**
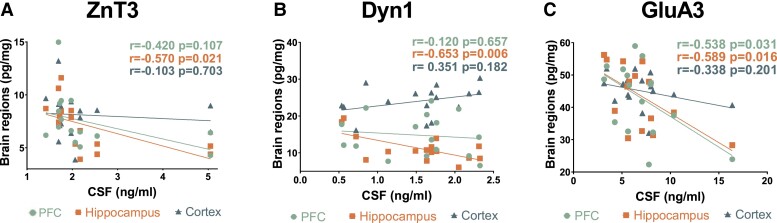
**CSF alterations correlate significantly with brain regional changes in *App* knock-in mice.** CSF concentrations of all three synaptic proteins correlated with hippocampal concentrations of the respective proteins (**C**, **B**, **C**), moreover CSF concentrations of GluA3 further correlated with PFC concentrations (**C**) Pearson’s correlation was used to determine associations. Abbreviations: CSF, cerebrospinal fluid; PFC, prefrontal cortex.

To further explore the potential of ZnT3, Dyn1 and GluA3 as synaptic biomarkers, we have exploited the unique possibility of a direct evaluation of brain regional (prefrontal cortex, hippocampus and cortex) changes and assess their reflection in CSF within the same *App^NL-F^*, *App^NL-G-F^* and *App^wt^* mice. Notably, all the three synaptic protein concentrations in the CSF correlated negatively with their corresponding concentrations in hippocampal lysates (Fig. 6A–C). Furthermore, increased CSF concentrations of GluA3 were also associated to decreased concentrations in prefrontal cortex (*r* = −0.538, *P* = 0.031, Fig. 6C). Additionally, concentrations of ZnT3 and GluA3 in prefrontal cortex correlated with hippocampal concentrations (*r* = 0.606, *P* = 0.016 for ZnT3; *r* = 0.630, *P* = 0.005 for GluA3, data not shown) as well as with cortical concentration changes (*r* = 0.546, *P* = 0.031 for ZnT3; *r* = 0.643, *P* = 0.007 for GluA3) (Supplementary Fig. 4).

## Discussion

Dementia is not part of normal aging, although it is undoubtedly more common in the elderly. Throughout the last decades intensified efforts have been devoted to disentangling the conundrum of pathophysiological and mechanistic changes leading to Alzheimer’s disease, to find disease modifying treatments. A glimpse of light came recently as the first medication, aducanumab, targeting the reduction of Aß has been recently approved by the US Food and Drug Administration. While several breakthroughs have been achieved in the past decades that increased our understanding of risk factors and have improved the early diagnosis and care of Alzheimer’s disease patients,^43^ there are several questions remaining to be answered regarding the impact of possible intervention and prospect to reverse the underlying pathology. The ability to measure synapse-specific biomarkers in CSF serves as a useful prognostic marker of disease progression for at-risk individuals (such as MCI patients) as well as an outcome measure for potential disease-modifying therapies and disease prognosis (i.e. drug efficacy in pharmaceutical trials).

The principal finding of this study is the elevated CSF concentrations of ZnT3 and GluA3 in Alzheimer’s disease and MCI patients versus healthy control subjects. Of great importance is the specific increase of CSF ZnT3 concentrations in the MCI-Cv versus MCI-nCv group of patients highlighting the prospective role of both ZnT3 and GluA3 in differentiating major neurocognitive disorder patients of the biological continuum of Alzheimer’s disease. In line with these findings, in a recent study, wherein we have compared Alzheimer’s disease, dementia with Lewy body and Parkinson’s disease with dementia patients to early preclinical patients with SCD who do not have any detectable objective cognitive or pathological alterations, we have found elevated ZnT3, Dyn1 and GluA3 concentrations. These increased CSF ZnT3 concentrations were associated with cognitive impairment in Alzheimer’s disease.^35^ While the individual diagnostic utility of these proteins was moderate when differentiating SCD patients from other dementias (∼0.7 AUC),^18^ control patients were differentiated with an excellent 0.8–0.95 AUC from Alzheimer’s disease or MCI in our current study.

Another significant finding of this study is the striking resemblance found in the CSF synaptic profile of the patient cohort and the *App* knock-in Alzheimer’s disease mice, especially the *App^NL-G-F^* mice that exhibit a stronger pathology compared with the *App^NL-F^* mice exhibit significantly elevated GluA3 and a trend towards increased Dyn1 concentrations in CSF. Interestingly ZnT3 concentrations were increased in the CSF of both MCI and Alzheimer’s disease patients. However, these changes were not mirrored in either *App* knock-in mouse model. One plausible explanation could be that whereas the CSF samples from patients derive from lumbar puncture, murine CSF is obtained directly from the cisterna magna. Another possible explanation is that even though the *App* knock-in mice harbour extensive Aβ plaque pathology, neuroinflammation and cognitive impairment a robust tauopathy is absent and may result in differing synaptic profiles. It is therefore tempting to conclude that ZnT3 and GluA3 alterations are linked to Aβ and pathways downstream of Aβ. However, the observed cortical and hippocampal levels of ZnT3, Dyn1 or GluA3 in the *App* knock-in models are highly comparable to previous observations of the synaptic profile of Alzheimer’s disease patients presenting along with the robust decrease in neurogranin, PSD95 or GluA3 levels region dependent changes in ZnT3 and Dyn1 levels.^19,21,44^

A very high translational predictability of an animal model is essential to envisage the outcome of a treatment more accurately in Alzheimer’s disease patients as well as to reduce the risk of preclinical drug response misinterpretation. More than 150 animal models, including drosophila, fish, murine and primate models have been generated to model the complexity of Alzheimer’s disease.^45^ Various mouse models of Alzheimer’s disease have been extensively studied to understand the underlying neurological changes and to test therapeutic strategies prior to clinical trials. While none of the animal models are yet able to fully reproduce all the aspects of such a multidimensional disease as Alzheimer’s disease, the *App* knock-in mouse models are in better concordance with the pathophysiological and clinical aspects of Alzheimer’s disease as the endogenous *App* expression pattern is fully preserved. Apart from the Aβ induced pathology, they also display neuroinflammation, synaptic alterations with age and genotype dependent progressive cognitive impairment.^29,34,46^ Studies in the Tg2576 and the 5xFAD transgenic mouse models of Alzheimer’s disease, have also reported reduced presynaptic and postsynaptic markers such as synaptophysin and PSD-95 at a similar extent as in *App* knock-in models.^34,47,48^ Faster disease progression models are more cost effective and thus often favoured in the field. Early alteration in the synaptic transmission and synaptic plasticity recorded in prefrontal cortex of three to four months old *App^NL-G-F^* mice has recently been reported and these changes later spread to hippocampal structures with increased intensity upon aging.^46^

Hippocampal and the prefrontal cortical regions were more closely investigated in our study based on their early involvement in Alzheimer’s disease, but also because of their essential functional interaction in the encoding and retrieval of working, emotional and episodic memory.^49^ Apart from the hippocampus, GluA3 is present throughout the cortical and subcortical structures^50^ suggesting its importance in synaptic plasticity. In addition to the changes observed in the hippocampal structures in the 24-month-old *App^NL-F^* mice, we have found a distinct decrease in its expression in the PC which is involved in higher-order processing of olfactory information, a vital sensory function for rodents. Olfactory dysfunction, the inability to store and recollect memories of smell, with identifying often incorrectly specific odours, is accounted to be one of the earliest symptoms in many Alzheimer’s disease patients.^51^ Interestingly, the olfactory deficit has been intensively studied as a possible marker for differentiating normal aging from Alzheimer’s disease and amnestic MCI with studies aiming to clarify its biomarker capacity as risk stratification for conversion to Alzheimer’s disease.^52^ The underlying mechanisms of olfactory dysfunction in Alzheimer’s disease pathology are not fully understood but Aβ plaques and synaptic interplay are being suspected as a key player.^53^ Our findings might offer an interesting complementary link to this mechanism.

Both pre and postsynaptic changes have been widely reported in Alzheimer’s disease as well as in animal models of Alzheimer’s disease, with a recent study reporting impaired turnover rate of presynaptic, in particular synaptic vesicle associated proteins in these knock-in mice^54^ highlighting the increased vulnerability of presynaptic vesicles. While ZnT3 has been shown to be crucial in spatial memory formation and certain behavioural properties^55^ the AMPA receptor GluA3 has only a marginal role in memory as the GluA3 knock-out animal exhibit mainly deficiencies in social interactions with strong, isolation-induced aggressive behaviour.^56^ In a recent study, the mRNA expression pattern of ZnT3, Dyn1 and GluA3 among others was systematically analysed in young and aged *App^NL-G-F^* mice using a combination of genome wide spatial transcriptomics and in-situ hybridization^57^ (https://alzmap.org). Consistent with our results, this revealed similar protein expression patterns with a marked age-related impairment in synaptic proteins in the aging *App^NL-G-F^* mice which was similarly observed at the mRNA expression level including the distribution pattern. Interestingly, an initial decrease in the mRNA levels of ZnT3 coding gene *Slc30a3* and *Dnm1,* coding for Dyn1, are observed at three months followed by an increase in cortical regions of 18-month-old *App^NL-G-F^* mice. Undeniably, more systematic studies are needed to reveal the dynamics of synaptic changes, nevertheless our results complement previous knowledge highlighting the similarities in alterations of synaptic vesicle related proteins observed both in early and mild Alzheimer’s disease patients and in *App* knock-in models.

While our approach of relating alterations of synaptic proteins in the brains of dementia patients to animal models frames the translational approach of this study, some limitations, deriving from the use of an animal model system for such a complex multidimensional human disease needs to be considered, while interpreting results. The *App* knock-in models have been shown to be free of the probable artefacts that transgenic mice generally suffer from. Nevertheless, these mouse models still have some limitations as they exhibit less Alzheimer’s disease-related tau pathology.^30^ Another shortcoming in our setup is the modest number of the memory clinic cohort and follow-up data of participants. For this study, we did not have the possibility to further increase the number of patients (especially with follow-up) included, we believe that replicating results with a longer follow-up period would add to the robustness of our results. A further limitation in interpreting the results is the inclusion of both sexes of animals which could affect the statistical power of the study. Female *App* knock-in mice show a stronger Alzheimer’s disease-like pathology than age matched male mice,^29^ but as the disease is affecting both genders, as reflected in the memory clinic cohort, striving towards the translatability of the synaptic changes found in Alzheimer’s disease patients, including each gender generalizes the conclusions of the results.

Taken together, our results suggest that the synaptic dysfunction observed in patients through the continuum of Alzheimer’s disease is of similar nature in the *App* knock-in mice as we have found high alignment in how the synaptic alterations are mirrored in these *App* knock-in mice. As research investigation of post-mortem neuropathological changes is often the primary objective of preclinical Alzheimer’s disease animal studies, with its translational value often overlooked, we have aimed to narrow this gap with our study.

## Conclusions and further directions

The elevated CSF ZnT3 concentrations in MCI-Cv versus MCI-NCv group of patients suggests a prospective role of ZnT3 in differentiating dementia patients of the biological continuum of Alzheimer’s disease. In addition, both Dyn1 and GluA3 may have diagnostic values. The pronounced synaptic alterations in MCI and Alzheimer’s disease patients were a discerned characteristic of the *App* knock-in mouse models which highlight the translational potential of these Alzheimer’s disease mouse models. Translatable synaptic biomarkers could potentially help reduce disparities between human and animal models-based studies aiding the translation of preclinical discoveries of pathophysiological changes into clinical research that could lead to meaningful new forms of therapy. Common translational research efforts to deepen our understanding of the disease mechanism as well as testing novel therapeutic strategies require adequate preclinical animal models that can accurately simulate complex human conditions, such as Alzheimer’s disease. These novel *App* knock-in mice exhibiting strong Aβ plaque pathology, neuroinflammation and synaptic dysfunction show memory decline that is likely to reflect aspects of the continuum of Alzheimer’s disease.^29^

## Supplementary Material

fcad001_Supplementary_Data

## Abbreviations

Aβ =: amyloid beta
App =: amyloid precursor protein
CSF =: cerebrospinal fluid
Dyn1 =: dynamin1
FSB =: 1-fluoro-2,5-bis(3-carboxy-4-hydroxystyryl) benzene
GFAP =: glial fibrillary acidic protein
GluA3 =: AMPA glutamate receptor 3
Iba1 =: Ionized calcium-binding adaptor molecule 1
MCI =: mild cognitive impairment
ZnT3 =: zinc transporter 3 protein
