# Supplementary material for: Translational potential of synaptic alterations in Alzheimer’s disease patients and amyloid precursor protein knock-in mice
Source: Brain Commun. 2023 Jan 5;5(1):fcad001. doi: 10.1093/braincomms/fcad001 (PMC9851419; doi:10.1093/braincomms/fcad001)
Supplement: fcad001_Supplementary_Data [file fcad001_supplementary_data.pdf]

Supplementary Figure 1

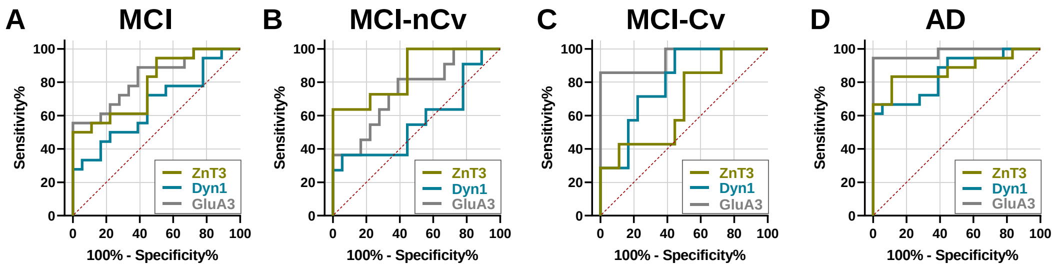

Supplementary figure 1: Diagnostic utility of ZnT3, GluA3 and Dynamin 1 in CSF of patients with various clinical dementias. Results from ROC curve analyses are visualized in panel **A-D** with area under curve, sensitivity and specificity of synaptic proteins. MCI: Mild cognitive decline; Cv: converters; nCv: non converters; AD: Alzheimer`s disease.

Supplementary Figure 2

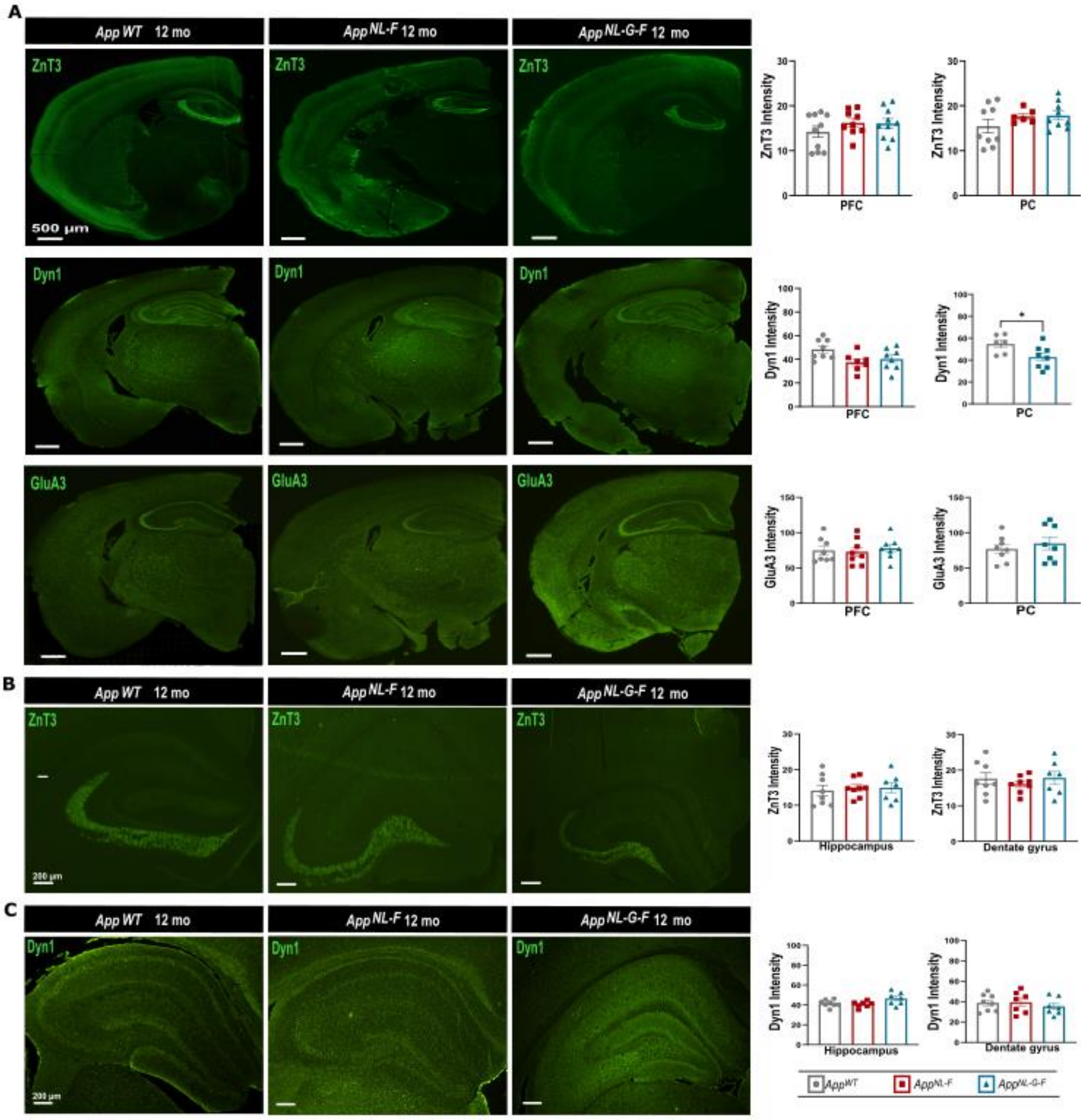

Supplementary Figure 2. Immunoreactivity of ZnT3 and Dyn1 in 12 months old *App* knock-in mice. (**A**) Overall immunoreactivity of Dyn1 revealed decreased immunoreactivity in the piriform cortex of 12-month-old App^{NL-F} mice with no further significant difference of cortical expression of the ZnT3 and GluA3 proteins of *App* knock-in mice compared to App^{wt}. No significant alteration in the hippocampal immunoreactivity of ZnT3 (**B**) nor Dyn1 was observed at 12 months (**C**). The differences between the groups were assessed using Kruskal–Wallis test followed by the Dunn multiple comparisons test. Abbreviations: PFC: prefrontal cortex, PC: piriform cortex.

Supplementary Figure 3

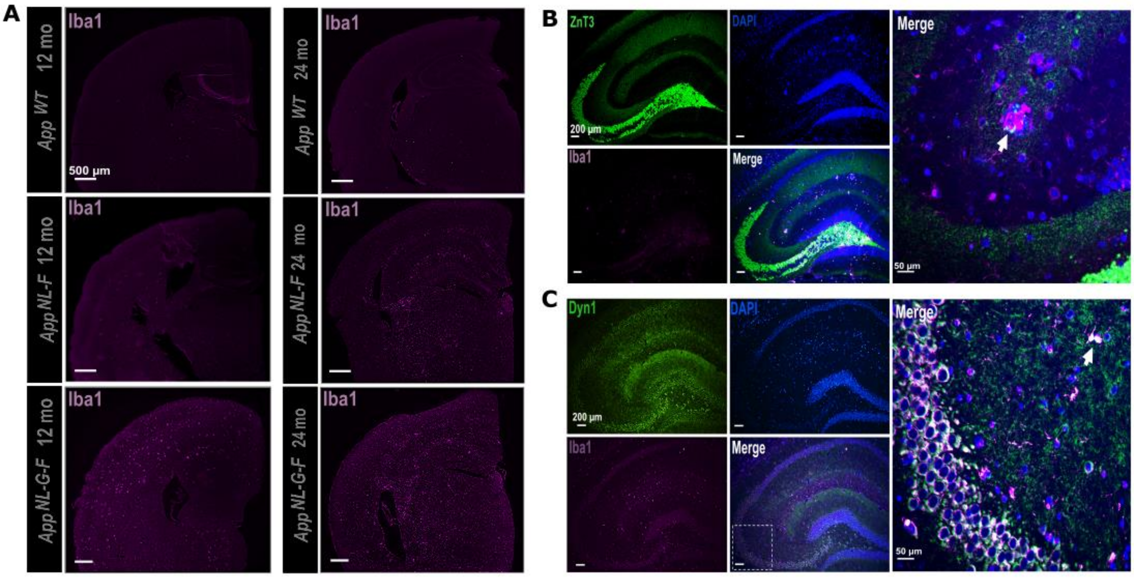

Supplementary Figure 3. Marked Iba1 immunoreactivity observed in *App* knock-in mice. (**A**) Marked accumulation of activated microglia revealed by Iba1(magenta) in 12 and 24-month-old *App*$^{NL-F}$ and *App*$^{NL-G-F}$ mice (**B**) Representative immunostaining revealing lack of co-localization between microglia marker (Iba1, red) and ZnT3 (green) in 24-month-old *App*$^{NL-G-F}$ mouse (**C**) Representative images showing no co-localization observed between microglia marker (Iba1, red) and Dyn1 (green) in 24-month-old *App*$^{NL-G-F}$ mice.

Supplementary Figure 4

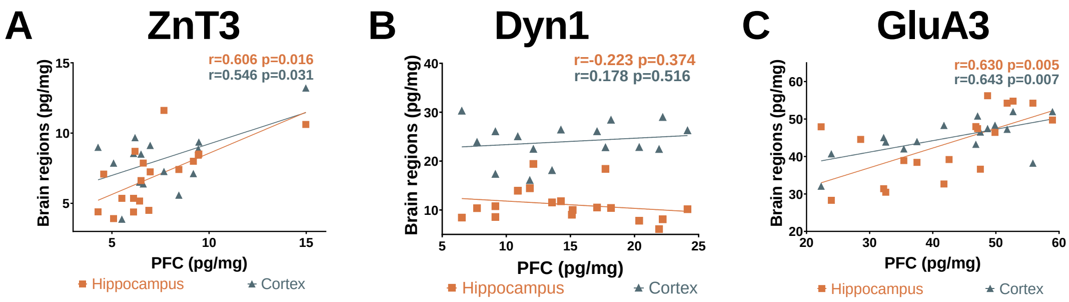

Supplementary Figure 4. Brain regional correlations of synaptic proteins in *App* knock-in mice. PFC concentrations of both ZnT3(**A**) and GluA3 (**C**) correlated with hippocampal and cortical concentrations in knock-in mice while no relationship was found in the caser of Dyn1 (**B**). Pearson`s correlation was used to determine associations. Abbreviations: PFC-prefrontal cortex.
